# Body Cosmos 2.0: embodied biofeedback interface for dancing

**DOI:** 10.1186/s42492-025-00207-9

**Published:** 2025-11-20

**Authors:** Rem RunGu Lin, Koo Yongen Ke, Kang Zhang

**Affiliations:** 1 Computational Media and Art, Hong Kong University of Science and Technology (Guangzhou), Guangzhou, Guangdong 510000 China; 2 Funtheory/Befun Lab, Guangzhou, Guangdong 510000 China

**Keywords:** Biofeedback, Embodied interaction, Electroencephalography visualization, Dancing technology

## Abstract

This study presents Body Cosmos 2.0, an embodied biofeedback system with an interactive interface situated at the intersection of dance, human-computer interaction, and bio-art. Building on the authors’ prior work, “Body Cosmos: An Immersive Experience Driven by Real-time Bio-data,” the system presents the concept of a ‘bio-body’-a dynamic digital embodiment of a dancer’s internal state-generated in real-time through electroencephalography, heart rate sensors, motion tracking, and visualization techniques. Dancers interact with the system through three distinct experiences “VR embodiment,” which enables them to experience their internal states from a first-person perspective; “dancing within your bio-body,” which immerses them in their internal physiological and emotional states; and “dancing with your bio-body,” which creates a bio-digital reflection for expressive development and experiential exploration. To evaluate the system’s effectiveness, a workshop was conducted with 24 experienced dancers to assess its impact on self-awareness, creativity, and dance expressions. This integration of biodata with artistic expression transcends traditional neurofeedback and delves into the realm of embodied cognition. The study explores the concept, development, and application of “Body Cosmos 2.0,” highlighting its potential to amplify self-awareness, augment performance, and expand the expressive and creative possibilities of dance.

**Supplementary Information:**

The online version contains supplementary material available at 10.1186/s42492-025-00207-9.

## Introduction

Dance is a powerful medium of human expression that translates emotions, thoughts, and stories into dynamic physical movement [1]. For dancers, fully capturing the essence of their performance relies on a deep awareness of the relationship between the body, movement, emotions, and the external environment. Yet in pursuing this awareness, many remain unaware of the complex inner signals of their biodata, including parameters (P) like brainwave activity. These physiological signals, which are often inducible, constantly evolve, reflecting the dancer’s cognitive and emotional states in real time.

Recent technological advancements combined with interdisciplinary collaboration have broadened the scope of this study [2–4]. The intersection of dance and cognitive neuroscience has facilitated partnerships between artists and scientists aiming to uncovering the subtle dimensions of human experience and expression [5]. In this collaborative context, biofeedback has emerged as a promising tool, offering a window into brain operations and providing dancers with immediate awareness of their internal bodily signals [6]. With the rise of accessible electroencephalography (EEG) headsets, heart rate sensors, and visualization technologies has enabled the creation of a ‘bio-body,’-an artistic embodiment that reflects a dancer’s physiological signals and physical movements.

**Fig. 1:**
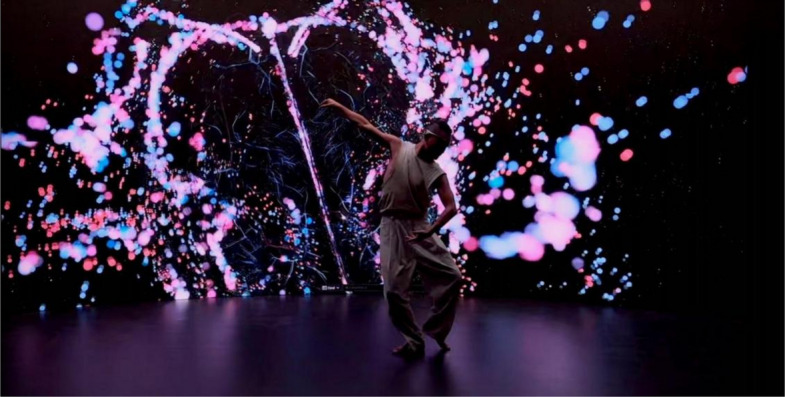
“Body Cosmos 2.0” is an embodied biofeedback system with an interactive interface designed for dancers to explore and interact with their internal physiological states in real time. Situated at the intersection of dance, human-computer interaction, and bio-art, the system combines advanced technologies to render biodata, such as brainwave activity, heart rate, and motion capture-into dynamic visualizations

Building on the authors’ prior work, “Body Cosmos: An Immersive Experience Driven by Real-Time Bio-Data” [7], this study introduces “Body Cosmos 2.0,” an evolved embodied a biofeedback system and an interactive interface designed to bridge the gap between the visible and the invisible in dance (Fig. 1). “Body Cosmos” explored the transformative potential of real-time biodata visualization in static and semi-dynamic settings. In “Body Cosmos 2.0,” the system extends this foundation by enabling full-body interactions and integrating new functionalities for dynamic and performative dance expressions.

This system introduces the concept of a “bio-body that integrates real-time biodata with motion capture and visualization technologies.” Through three unique interactive experiences: “virtual reality (VR) embodiment,” which enables dancers to experience their internal states from a first-person perspective; “dancing within your bio-body,” which immerses dancers in their internal landscape; and “dancing with your bio-body,” which creates a bio-digital reflection for performance enhancement and interactive exploration. The system aims to foster kinaesthetic creativity and somatic self-awareness.

To evaluate the effectiveness of the system, a dancing workshop was conducted with 24 experienced dancers. This workshop assessed the impact of “Body Cosmos 2.0” on self-awareness, creativity, and dance expression, providing empirical insights into its applications and benefits. By combining the dancer’s accumulated knowledge and training with instantaneous feedback from their body, the system opens avenues for more responsive and performative dance expressions. This integration of biodata with artistic expression positions “Body Cosmos 2.0” as a bridge between human cognition, creativity, and digital technology, offering new possibilities for dynamic and adaptive artistic processes.

The primary contributions of this study are as follows:

- An embodied biofeedback system and interactive interface that introduces a new approach to conceptualizing, interacting with, and expressing dance. This perspective fosters a dynamic and adaptive artistic process that extends dance’s expressive capabilities by merging movements with emotional and cognitive expressions.
- A systematic approach to creating a bio-body, a medium that integrates real-time biodata with artistic expressions for dancers. It offers a tool for dancers to engage deeply and responsively with their internal state, thereby enriching the expressiveness of their performance.

### Background

#### Somatic turn in human computer interaction

In the third-wave human computer interaction (HCI), the body is viewed as an active agent of sensing, experiencing, and expressing, intricately embedded in its surrounding context [8]. This perspective aligns with Merleau-Ponty’s phenomenology, which places the human body at the center of perception and action, emphasizing that bodily experiences shape our understanding of the world [9]. Notable works in this domain include”The Lived Body in Design” [10],”Transferring Qualities from Horseback Riding to Design” [11],”Embodied Sketching” [12],”Move to Get Moved” [13], and a series of studies on bodily play [14–16].

This paradigm has evolved from an initial focus on bodily movement to a broader exploration of the body’s multimodal forms of communication [17]. Among these sensory modalities, kinesthesia and proprioception are particularly significant as they provide kinesthetic awareness and creativity, enabling the generation of novel movements and fostering a deeper connection between the body and mind [18].

The concept of ‘soma,’ which refers to the interconnectedness of the mind, body, and emotions, is central to our research [19–21]. This shift from viewing participants merely as ‘users’ to recognizing the integrated ‘body-mind’ experience is known as the “somatic turn” in HCI [22]. This perspective moves beyond the traditional user-centered approach, focusing instead on the holistic experience of the body as both a sensing and expressive entity.

As the foundation of our perception [9], the body is a vital tool for self-awareness, creating a feedback loop between bodily movements and emotional states [18]. This self-awareness feedback loop, which is essential for embodied interaction, is a key aspect of our study, allowing participants to continually adjust and refine their movements based on both internal states and external stimuli [23].

#### Dance and technical artifacts

Dance is a prominent form of embodied interaction [18]. Researchers have used dance to study body movements in interaction design, emphasizing the role of the body as a medium for perception, experience, and expression [24]. Improvisational dance, in particular, employs full-body perception and movement, integrating spontaneous actions into an active learning process [25].

What sets dance apart from other areas in HCI is its focus on exploring abstract meaning through embodied experience [24]. Embracing this “openness for interpretation” [26] opens up alternative ways to integrate technology into the nuanced and abstract processes of dance creation [27]. Kinesthetic creativity is defined as the body’s ability to generate metaphors, tell stories, and foster self-expression [8]. This form of creativity highlights the intrinsic link between physical movement and cognitive processes, whereby the body’s actions can reflect and influence emotional and mental states.

The exploration of dance within HCI involves the use of technology to enhance and analyze dance performances. This includes two key approaches: directly capturing dancers’ bodily data via a range of sensing technologies, referred to as “reading the body,” and cultivating the dancer’s multisensory awareness and perception, known as “writing the body” [24].

Advancements in media and sensing technologies have enabled researchers to capture dancer movements using motion capture systems [28] and physiological data, such as muscle activity [29]. Researchers have utilized various technological artifacts to enhance the dance experience, such as interactive costumes [30], sonification [31, 32], visualization technologies [33, 34], and the integration of collaborative agents, including robots [35], drones [27, 36], and artificial intelligence agents [37].

#### Biofeedback in interactive art

The integration of biodata, e.g., brainwave activity, into art has not been a recent initiative. Pioneering experiments with artistic brain-computer interfaces (BCIs) in art can be traced back to the 1960s, with landmark works like Music for a Solo Performer by Alvin Lucier [38]. However, advancements in technology, especially the increased accessibility of low-cost BCIs and software development kits (SDKs), have inspired artists and researchers across various disciplines to incorporate neural activity into diverse forms of art, including audio, visual, immersive, installation, and performance [39].

Notable work in this domain includes You Are the Ocean, which encourages participants to become aware of their internal states by demonstrating their impact on the external environment and fostering a sense of self-augmentation [40]. Similarly, Park’s Eunoia used BCIs to create dynamic, responsive environments and convert mental states into sound vibrations affecting the movement of water-revealing the intricate link between inner cognitive processes and outward physical manifestations [41].

In addition to self-augmentation, the BCI-based art explores the inner self. For example, Life Ink (Ars Electronica Futurelab) transforms human brainwaves and body signals into a dynamic stream of three-dimensional (3D) “Life Ink,” visualizing creativity as an external expression [42]. Similarly, Bio-Ink is a generative artwork inspired by the traditional eastern concept of qi. It combines EEG, electromyography, and motion data to create a fusion of internal representations and cursive calligraphy, thereby representing the inner state of the calligrapher through a particle-based system [43].

In the domain of dance, biofeedback has been employed to investigate the relation-relationship between physiological state and artistic expression. Raymond et al. [44] explored how real-time physiological monitoring could enhance dancers’ performance. Gruzelier et al. [2] demonstrated the potential of biofeedback training in boosting creativity and reducing state anxiety in contemporary dancers, thereby fostering optimal performance. More recently, Lin et al. [45] introduced an intracorporeal biofeedback interface designed for contact improvisation that enabled participants to engage in shared somatic experiences through dynamic feedback.

#### Our innovation

While these works have significantly advanced the fields of embodied interaction, dance, and biodata in art and performance, existing systems often focus on unidimensional feedback mechanisms and lack the full-body integration that characterizes our approach. Similarly, wearable devices and interactive costumes have provided dancers with biodata-driven feedback; however, they typically emphasize localized or fragmented signals rather than offering a holistic representation of a dancer’s body.

In contrast, “Body Cosmos 2.0,” which distinguishes itself by integrating real-time EEG, heart rate, and motion capture data, creates a unified body experience that is both immersive and interactive. By offering three distinct interactive experiences: the VR embodiment, dancing within your bio-body, and dancing with your bio-body, the system extends beyond the static or semi-dynamic visualizations seen in prior work. By combining self-augmentation, self-insight, and an embodied biofeedback loop into a unified system, “Body Cosmos 2.0” introduces a continuous embodied feedback cycle in which biodata dynamically influences dance movements, and these movements, in turn, shape biodata visualization. This approach expands the expressive potential of biofeedback systems by fostering deeper connections among physiological states, movements, and artistic expression.

Furthermore, while previous studies have often confined their exploration to controlled laboratory settings, “Body Cosmos 2.0” is designed with performative contexts in mind. Its adaptability to full-body movements and immersive environments underscores its potential for use in live performances and interactive dance training. The synergy between biodata integration, multimodal interaction, and real-time responsiveness underscores the value of our system, positioning it as a transformative tool that bridges human cognition, creative expression, and digital technology.

### Conceptual framework

#### From biofeedback to embodied biofeedback

**Fig. 2:**
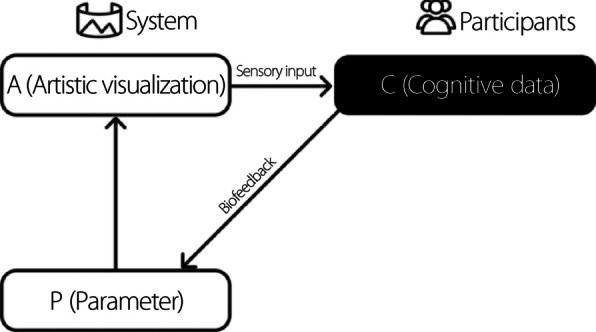
The neuro-feedback loop connects the A, perceived through sensory input (e.g., sight and sound), with the individual’s C, recorded via bio-sensors to modulate real-time P that influence the A. This iterative process creates a dynamic interaction between the viewer and the A

Traditional biofeedback in art is often represented as a simple, cyclic process: an evolving artistic visualization (A) is experienced by individuals through their sensory channels, which in turn triggers cognitive data (C) and immediate perceptual and decision-making responses to artistic stimuli (Fig. 2). In this conventional model, biosensors capture raw physiological signals such as brain activity (EEG) or heart rate. These biodata are then used to modulate the P in real time, thereby affecting the generative aspects of A and completing the A-C-P-A loop.

Our work builds on this traditional loop by adding an interpretative layer: artistic knowledge (K). Unlike raw biodata, K reflects a dancer’s long-term expertise and encompasses art theory, aesthetics, cultural contexts, and experiential insights. This additional layer informs how the dancers interpret both As and their own biodata. In our framework, the relationships among the components are structured as follows:

1. C: Immediate perceptual and decision-making responses generated by the dancer who experiences A and interacts with the system.
2. K: Dancers’ accumulated expertise, including their understanding of art theory, technique, aesthetics, and cultural influence.
3. Artistic exploration (E): Creative decisions and expressive movements emerge when C are enriched with K. This exploration is dynamic and personal in nature.
4. P: The system maps the integrated output of the biodata and body movement onto dynamic P that continuously modulate A, resulting in meaningful visual transformations.

**Fig. 3:**
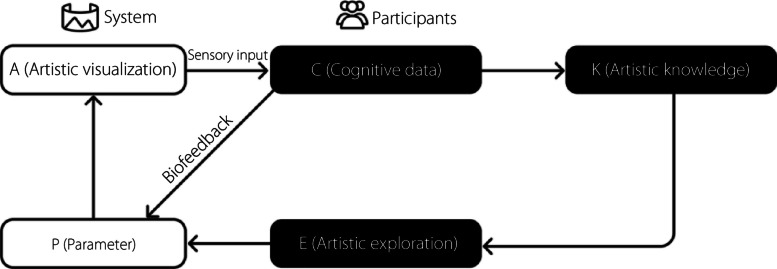
The transition from neuro-feedback to embodied cognition: A, influenced by P via bio-sensors, is shaped by the dancer’s C, K, and E. This expanded loop emphasizes the interplay between body, mind, and artistic output

In this expanded framework (Fig. 3), the process unfolds as follows: The dancer’s sensory experience generates immediate C, which are then enriched by their long-term K. This integration leads to E and the dancer’s creative and expressive responses. Simultaneously, the biosensors capture biodata that reflect the dancer’s physiological state. The system combines these layers and maps them onto P that govern evolving A. The resulting embodied biofeedback loop, A-C-P-A and A-C-K-E-P-A, ensures that A is continuously transformed by physiological signals, but also by the dancer’s deeper learned artistic sensibilities.

Although integrating K into body data is an ambitious goal, algorithmically deriving K from movements remains a significant challenge. The current work presents only the initial approaches that capture a limited subset of the rich nuances inherent in artistic expressions. Future iterations should refine these methods by incorporating qualitative data, advanced machine learning techniques, and multimodal inputs to represent a dancer’s artistic insights more accurately and strengthen the integration from K to E to P.

Drawing on Varela’s theory of the embodied mind [46] and Merleau-Ponty’s notion of embodied perception [47], cognition is understood not as a purely brain-centered activity but as a process that arises from the body’s dynamic interactions with its environment. In this context, dancers’ K informs their E-the creative decisions and movements they make during the artistic process. This E then intertwines with the P, dynamically shaping and influencing the output of A [48]. This expanded framework emphasizes the interplay between mind, body, and environment, showcasing how embodied cognition drives creativity and artistic expressions.

Merleau-Ponty’s phenomenological approach underscores the significance of the body as the primary site for knowing the world, highlighting how perception and action are intrinsically linked [47]. Extending biofeedback to embodied biofeedback confirms that dancers’ bodily experiences and movements are fundamental to their cognitive and creative processes. The embodied biofeedback loop in “Body Cosmos 2.0” captures physiological data and resonates with the dancer’s lived perceptual experiences, deepening the connection between internal states and expressive movements.

#### The bio-body: between virtual body and cyber-body

The bio-body concept occupies an intermediary space between the virtual body and cyber-body, embodying a unique fusion of biological data and digital technology. This integration resonates deeply with Marshall McLuhan’s assertion that “the medium is the extension of man” [49], suggesting that technology not only augments our physical capabilities but also extends our internal awareness, as further elaborated by Rowland [50]. This perspective transcends the mere physicality of the human form by integrating an individual’s inner consciousness, thereby positing personal awareness as a medium.

Rooted in cyberculture, the cyber-body epitomizes the integration of humans with digital technology, such as VR, challenging traditional boundaries between humans and machines and the self and others [51, 52]. It embodies a form of digital resistance and post-humanism, as articulated in Haraway’s Cyborg Manifesto [53], emphasizing the potential to transcend biological limitations through technological augmentation.

Based on computational modeling, a virtual body offers an astable and measurable representation of human anatomy and physiology [54]. It often reflects prevailing cultural norms and ideals, translating the human form into a digital performance entity [55]. A virtual body is typically designed for consistency and replicability, for example, reconstructed from magnetic resonance imaging (MRI), and serves as a tool for simulation, training, or aesthetic purposes in digital environments.

In contrast to both the cyber-body and virtual body, the bio-body represents a dynamic nexus where real-time physiological data and digital feedback coalesce. This transcends the static augmentation of the cyber-body and fixed representation of the virtual body by enabling a mutable and adaptable entity that continuously evolves based on the physiological and emotional states of the dancer. This dynamic interaction allows the bio-body to serve as both a digital mirror and interactive medium that responds to and influences the dancer’s movements and internal states.

##### Data vs flesh

The bio-body epitomizes the convergence of data and flesh, where biological signals (flesh) are transformed into digital inputs and outputs. Unlike a virtual body that offers a stable and often idealized digital representation [54] or cyberbody, which emphasizes augmentation and extension through technology [51], the bio-body creates a symbiotic relationship in which real-time physiological data directly influences and is influenced by digital representation. This synthesis blurs the boundaries between the organic and digital, fostering an interactive and responsive interface that reflects the dancer’s current physiological and emotional states.

##### Input vs output

The bio-body operates as an input and output medium, facilitating the bidirectional flow of information. Biosensors capture detailed physiological data such as EEG, heart rate, and motion, and serve as inputs that modulate P in real time. Concurrently, the system outputs visual and interactive responses to which dancers can perceive and react instantaneously. This dynamic feedback loop enhances self-awareness and performance, creating a continuous dialogue between the dancers’ internal states and their expressive movements.

##### Human vs technology

The integration of human physiological data with technological visualization tools exemplifies the blending of humans and machines. Unlike the cyber-body, which often presents technology as an external enhancement, or the virtual body, which maintains a clear distinction between human and digital elements, the body emphasizes a symbiotic relationship in which technology expands the dancer’s body and consciousness. This deep interdependence positions the bio-body as a bridge between the corporeal and digital, enabling the integration of human and technological elements within the dance realm.

## Methods

### Designing “Body Cosmos 2.0”

#### Relationship between the bio-body and the dancer

We propose the concept of a bio-body for a dancer that integrates their movement, expression, cognition, and artistic expression. A bio-body is a dynamic and adaptive entity that evolves through bodily exploration and cognitive feedback. The relationship between the body and participant is illustrated in Fig. 3.

A dancer’s K is their understanding of techniques, culture, aesthetics, and emotions. This knowledge informs and guides artistic choices and expressions. The dancers’ K is based on their prior learning and experience, as well as their current context and goals.

A dancer’s E is a body movement encoded by emotion and expression. This exploration was captured using a webcam through computer vision and transformed into P. K influenced dancers’ E.

Dancers’ C is their ability to respond to artistic elements and emotions or inner states generated by A. C is affected by visual feedback from A in real time. The EEG headband and heart rate sensor recorded this C and used it to adjust the P in real time, creating a feedback loop between the dancer and the A.

The P are the position and rotation of the bones of the skeleton and P such as brightness, noise, color of the shaders, and particle systems.

A is an evolving or final art piece generated by P. It also reflects the dancer’s E and embodies dancer’s C.

#### Compositions of the bio-body

The bio-body is composed of the following elements:

Particle systems: These simulate the brain, heart, and blood vessels based on digital imaging and communications in medicine (DICOM) data. Particle systems are controlled by P influenced by the real-time biodata captured by the dancer. Procedurally generated models: These models simulate the nervous system and are controlled by P influenced by real-time biodata captured from the dancer. Bio-responsive shaders: Customized shaders are designed to enable parallel systems and procedural models to modify mesh vertices and UV P in response to biological data inputs. Proxy Skeleton: This skeleton drives the movement of the body and is driven by the dancer’s movements captured through motion-capture technology.

We used an EEG headband and heart rate sensor to record the dancer’s C and adjust the P in real time. The dancer’s motion and movements were captured using a webcam and computer vision, thereby influencing the P in real time. The P include the position and rotation of the bones of the skeleton and attributes such as the brightness, noise, and color of the shaders and particle systems. A is generated by these P, reflecting the dancer’s E and embodying the dancer’s C.

#### Particle system

DICOM data are a common format for medical imaging such as computed tomography (CT) or MRI scans, which provide a window into internal organs and structures [56]. However, traditional methods that use such data, such as volumetric rendering, produce static 3D images that lack interactivity and dynamism. We propose an approach that transforms static DICOM data into interactive 3D visualization by incorporating a particle system that reacts to dancers’ real-time biodata. Houdini software was used to transform the DICOM slices into 3D mesh models, which were subsequently brought into Unreal Engine 5 to serve as the foundation for the particle emitters. This technique creates a visual dialogue between dancers’ physiological states and digital representations.

**Fig. 4:**
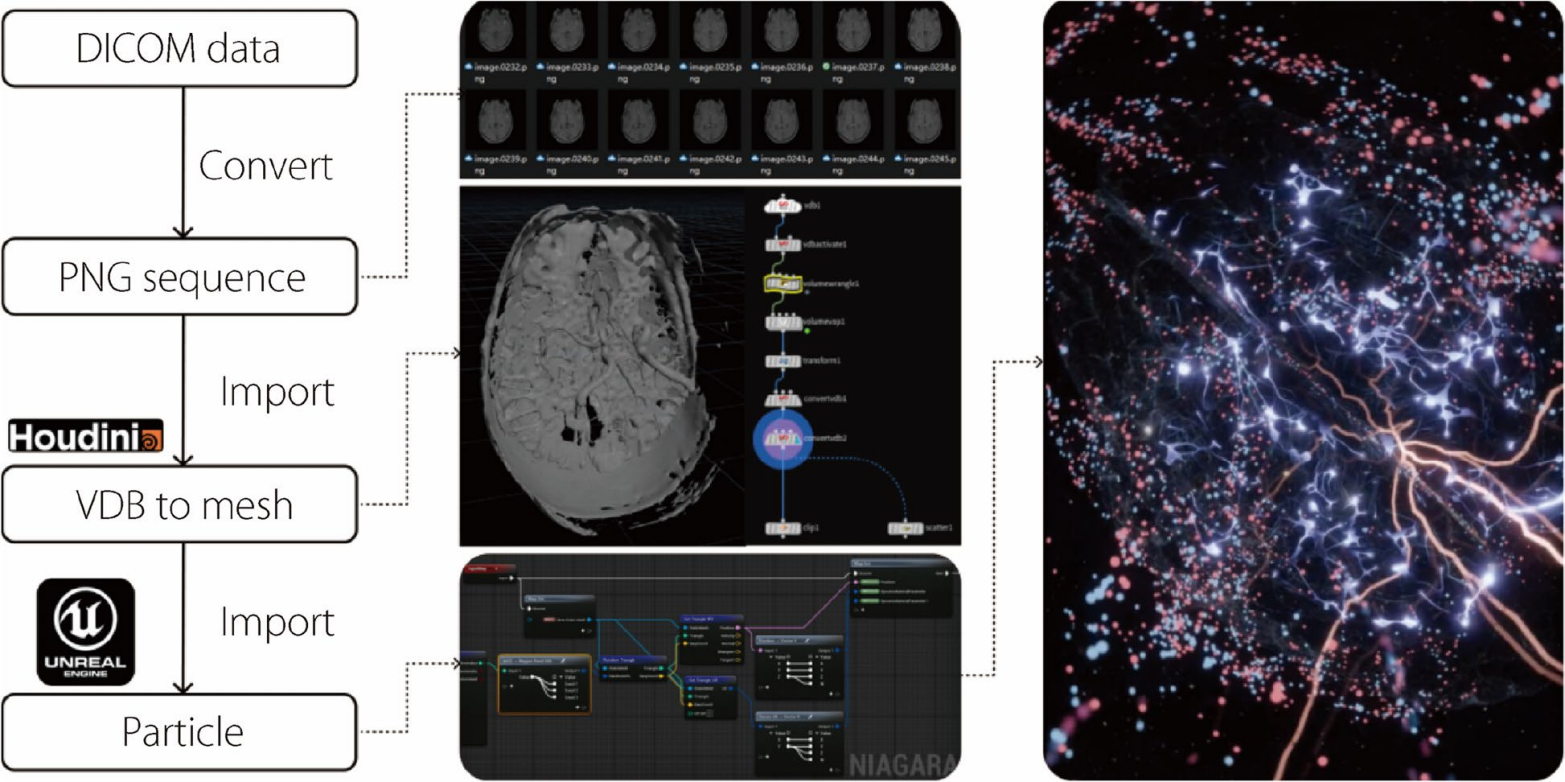
Particle system based on DICOM data

To illustrate our approach, we provide a brain visualization that dynamically reacts to the user’s brainwaves by altering its color, noise, and brightness. The process of crafting this brain visualization is outlined as follows (Fig. 4):

- We start by sourcing DICOM scans of the human brain from the Natural Scenes dataset [57] and transforming them into a sequence of portable network graphics images using an online conversion tool.
- The image sequences were imported into Houdini, where we created a voxel data block (VDB) with an assigned density attribute.
- Within Houdini, a Volume Wrangle node was employed to write Vector Expression Language scripts. These scripts correlated the length of the image sequences with the *z*-axis depth of the VDB. We utilized a color map function within this node to interpret the color information from the image sequences and convert it into the corresponding density metrics for the VDB. To further enhance the visual clarity of the VDB, a volume VOP node was applied to introduce a contrast ramp, amplifying the distinction between density regions. After these preprocessing steps, the VDB was converted into a 3D mesh and transferred into Unreal Engine 5 for real-time visualization.
- Within Unreal Engine 5, the Niagara system was employed to generate a dynamic particle display across the surface of the 3D mesh. To enable real-time responsiveness to physiological data, we developed specialized shaders capable of deforming mesh vertices based on biodata input. These shaders were built with interfaces that directly receive signals from an EEG system and are programmed to utilize both the mesh’s world position and UV coordinates imported from Houdini, allowing for continuous visual adjustments driven by real-time data.
- Finally, we used the EEG data to modulate the attributes of the particle system, specifically influencing the color, noise, and brightness, to visually reflect the brain’s activity.

#### Procedurally generated models

To construct the nerve model within the bio-body, we leveraged the power of procedural modeling in Houdini. This process allows the creation of a nerve system mesh that is anatomically representative and intricately detailed. Procedural modeling also enabled us to create a UV (horizontal and vertical texture coordinates) map that accurately reflects the 3D structure onto a two-dimensional texture space. This is crucial for texturing and controlling flowing light effects, which will later be applied in a real engine.

**Fig. 5:**
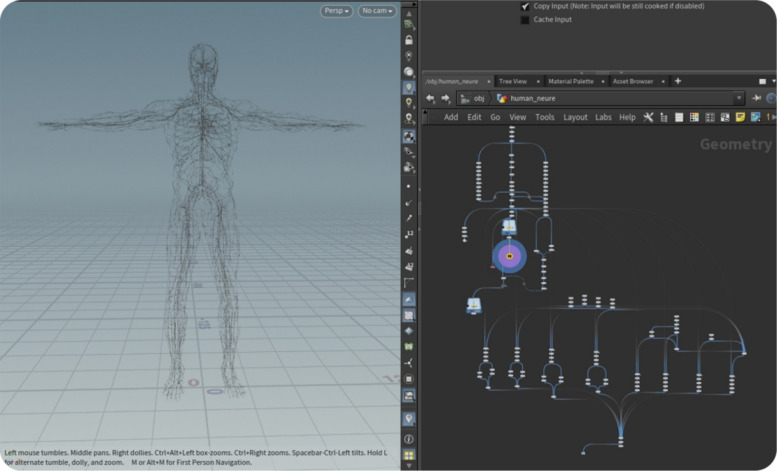
The mesh of the nerve generated by procedural modeling

Outlined below is the process for devising the nerve mesh (Fig. 5):

- The process begins with the definition of the nervous system’s fundamental structure. This is achieved by establishing a series of points or nodes within the Houdini that represent the junctures of the neural pathways.
- A noise function was applied to introduce natural variations into nerve pathways, creating a more organic representation. This function adds irregularities and diversity to the proposed model.
- We generated a UV map that translates the 3D structure into a two-dimensional plane. This map is essential for the later application of textures and effects in real engine environments.
- The nervous system mesh and its corresponding UV map were imported into UE5. Customized shaders are created to receive biodata inputs, such as EEG and heart rate information, to control the visual effects in real time.
- These shaders are designed to manipulate the flow and intensity of light along nerve pathways. As a specific indicator, when coherence reaches a certain level, it influences the shader, altering the effects of light to mirror the dancer’s physiological signals.

#### Bio-responsive shader

The intricate particle systems and procedural models in our “Body Cosmos 2.0” project were powered by specialized shaders designed to dynamically respond to biological data inputs. These shaders are adept at modifying the mesh vertices and UV P in real-time, creating visually compelling transformations based on physiological states. Shaders are customized to integrate seamlessly with biodata inputs, enabling the system to reflect cognitive and emotional changes as they occur.

The core of this system is a flowtime EEG headband, which includes a heart rate sensor [58]. The EEG headband measures brainwaves (α, β, θ, δ, γ) using eight sensors capturing signals from the left and right hemispheres. Operating at a bandwidth of 250 Hz, it detects brain activity ranging from -2 µV to 2 µV. These raw signals were transmitted via Bluetooth to a local Python-based application that leverages FlowTime’s SDK [59] for initial preprocessing.

In this application, the EEG data underwent filtering and spectral analysis to isolate the relevant frequency bands. The proprietary algorithms in FlowTime’s Emotion Cloud process these frequency components to derive key emotional indicators, including attention, relaxation, pressure, and coherence, each ranging from 0 to 100. These indicators are updated approximately every 600 ms and transmitted back to the local server. From there, the processed data are converted into open sound control (OSC) messages and sent to the Unreal Engine via the user datagram protocol. The shaders and particle systems in Unreal Engine adjust dynamically in response to these incoming data streams, creating a real-time connection between the dancer’s physiological states and visual outputs.

Within Unreal Engine, specialized shaders and particle systems interpret these OSC messages to dynamically adjust the visual P. For example, changes in the dancer’s physiological signals were mapped to various visual effects (Table 1).

**Table 1:** Interactive rules with the real-time bio-data

Sensor	Indicator	Bio-nebula	P	Relationship
Heart rate sensor	HR	Heart nebula Vessel nebula	Frequency velocity	
EEG headband	Attention	Brain nebula Nerve nebula	Emissive intensity	
	Relaxation	Brain nebula Vessel nebula	Noise frequency	
	Pressure	Brain nebula	Scale color

- Heart rate: Controls the frequency of pulsation and the velocity of moving visual elements (the ‘vessel’), with increased heart rates amplifying both attributes.
- Attention indicator: Brightness of key visual elements (the ‘brain’ and ‘nerve’ and nerves), where higher attention levels yield more intense illumination.
- Relaxation indicator: Modulates the noise frequency within the visual patterns of the brain and vessels, producing smoother patterns with higher relaxation.
- Stress indicator: Influences the color gradient, shifting from calming blue to intense red as stress levels increase.

This integration creates a direct, real-time connection between the dancer’s physiological state and the visual output. By translating raw biodata into dynamic shader P, the system reflects changes in cognitive and emotional states (as indicated by EEG and heart rate data) and enriches these transformations through the dancer’s inherent artistic insights.

#### Proxy skeleton

The procedural model and particle system mesh of the bio-body, characterized by its extensive polygon count, present challenges for traditional animation techniques such as skeletal binding and skinning. To address this, we employed an approach from Houdini using a basic skeletal structure to drive a proxy model, which in turn controls the bio-body. This method facilitates the efficient manipulation of a complex bio-body mesh, including nerve and vascular systems.

**Fig. 6:**
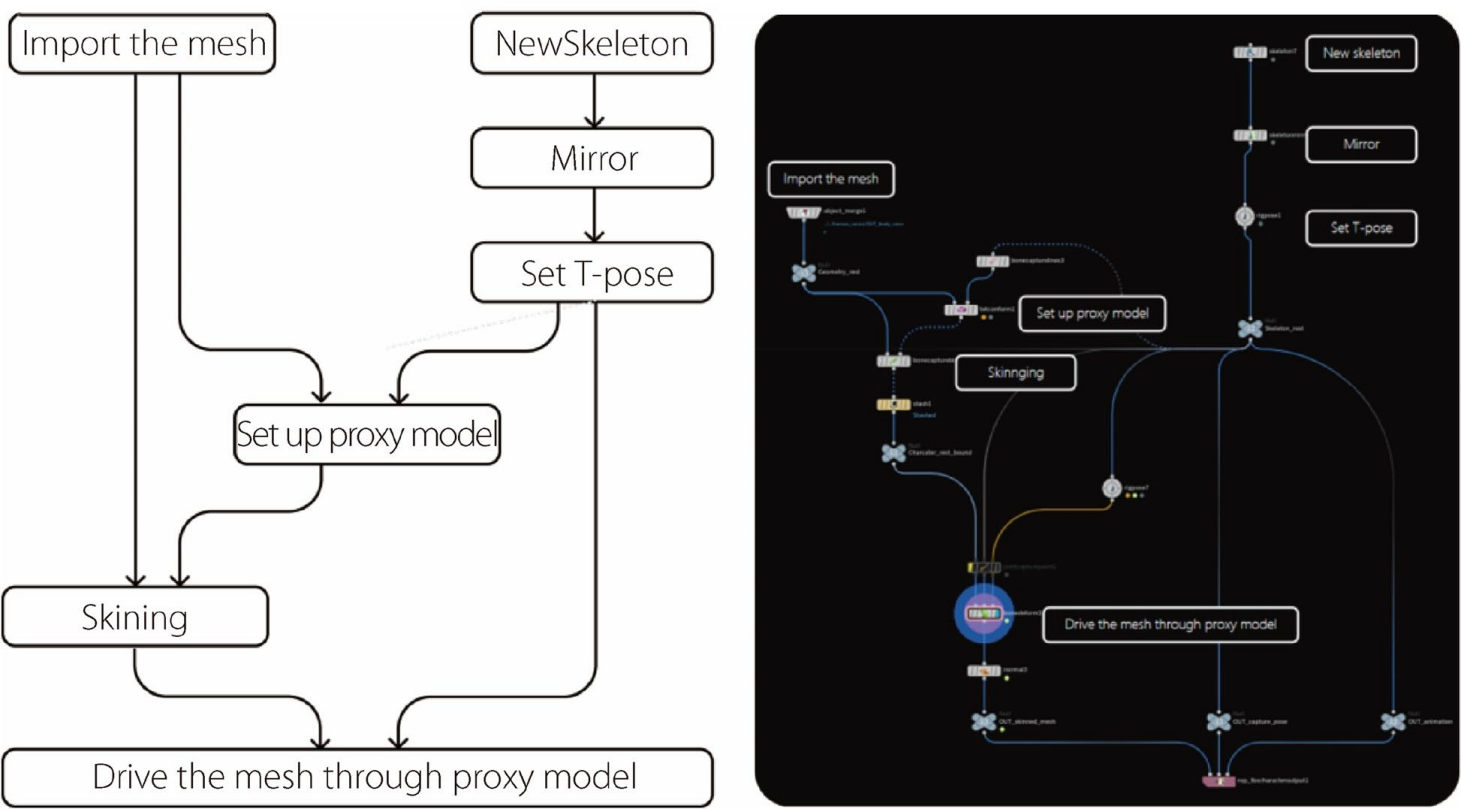
The process of creating a proxy skeleton

The process unfolds as follows (Fig. 6):

- In Houdini, the foundational skeletal structure was initiated. This skeleton acts as the core framework upon which the movements of the body hinge.
- A proxy model that is simplified yet structurally representative of the bio-body is driven by this basic skeleton. This proxy model acts as an intermediary, reducing computational load while accurately conveying the intended movements to the body.
- Subsequently, the bio-body mesh, now rigged with the skeleton, was exported. This mesh includes detailed representations of the neural and vascular systems and is rigged to ensure movement and deformation.
- After export, they were imported to Unreal Engine. Within the Persona module of the engine, the heart and brain meshes were attached to skeletal joints using sockets. The engine then computes the offsets of these meshes relative to their corresponding skeletal joints to ensure accurate alignment and movement.

To capture the dancer’s movements, we integrated the Dollers MoCap software [60]. This tool uses a webcam and leverages computer vision technology to facilitate real-time motion capture. This process is not only about tracking physical movements but also integrating the dancer’s K into the body’s response system. As the dancers’ movements, which embody their E, are captured and digitally translated, the system contextualizes these movements within their broader artistic framework. Thus, the real-time motion data were converted into P, including the positions and rotations of the skeletal bones. This approach ensures that the bio-body mirrors the dancer’s physical movements and reflects their artistic intent and expression, facilitating a blend of the physical and digital realms and enhancing the depth and meaning of the artistic representation.

### Mode of experience

“Body Cosmos 2.0” features three distinct interaction modes: VR embodiment, dancing within your bio-body, and dancing with your bio-body. Each mode offers a distinct perspective and interaction, intertwining dance, digital expression, and the exploration of cognition and creativity.

#### VR embodiment

The VR embodiment offers an immersive environment through which dancers can explore their internal states from a first-person perspective. Through the use of VR, dancers are transported into the body’s cosmos, a digital representation of their physiological and cognitive states, providing a deep connection to the dancer’s biofeedback loop in a cosmic environment.

In this mode, dancers wear VR headsets that visually embody them in the virtual body. The VR experience is synchronized with real-time biometric data, such as heart rate and brain activity, allowing dancers to perceive and feel their physiological states immersively.

#### Dancing within your bio-body

**Fig. 7:**
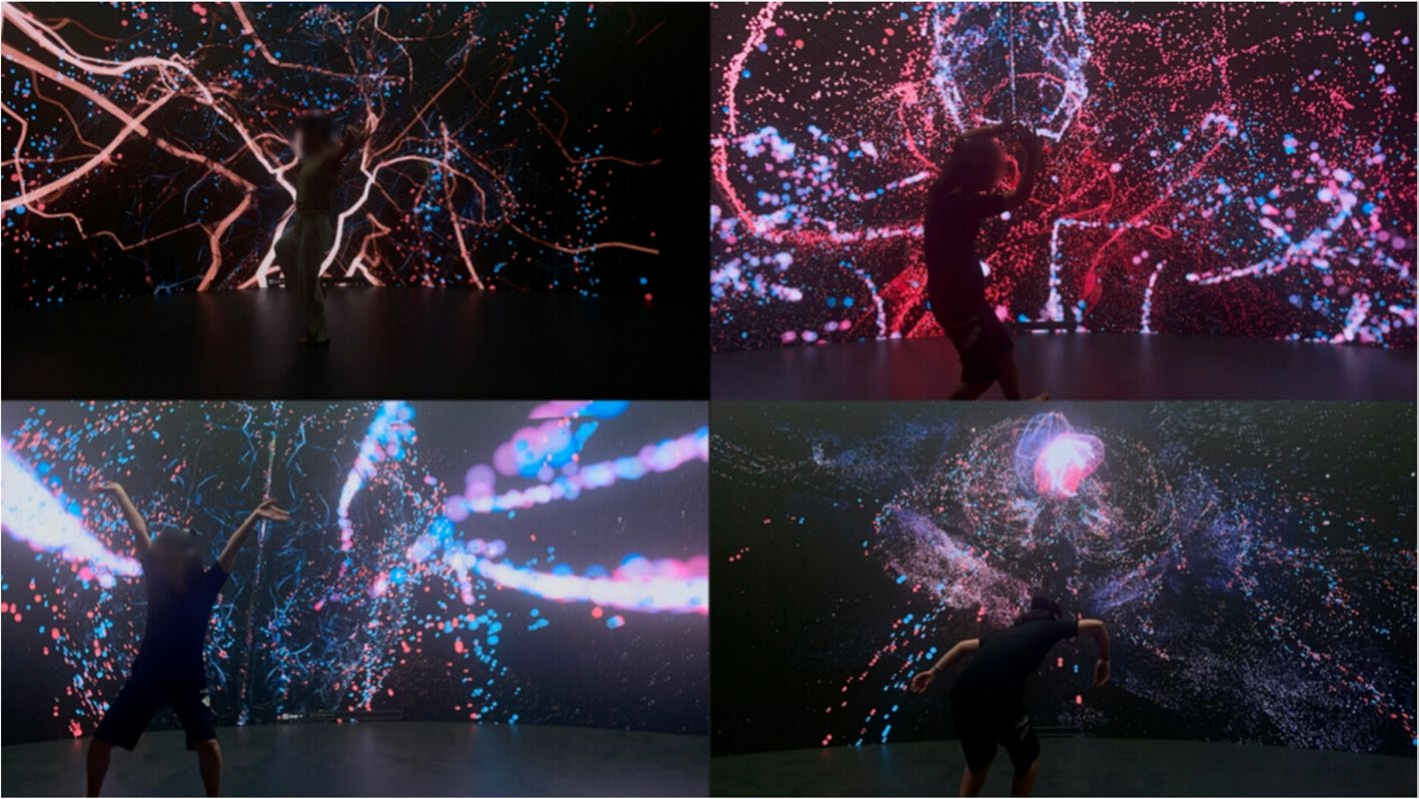
Dancing within your bio-body

Dancing within our bodies offers an immersive experience in which dancers metaphorically journey into their own physiological space (Fig. 7). This mode employs a customized camera trajectory to traverse a digitally reconstructed bio-body, providing a unique view on the dancer’s internal state.

Wearing an EEG headband and heart rate sensor, dancers had their cognitive focus and emotional responses continuously tracked. The biometric input collected in real time shaped the visuals rendered on a curved LED display. This immersive environment placed the dancer inside their own physiological landscape, forming a feedback loop where inner states actively influenced the visual output. Such interaction exemplifies principles of embodied cognition, wherein bodily actions modulate mental processes [46], and highlights the deep interconnection between mind, body, and digital expression.

#### Dancing with your bio-body

**Fig. 8:**
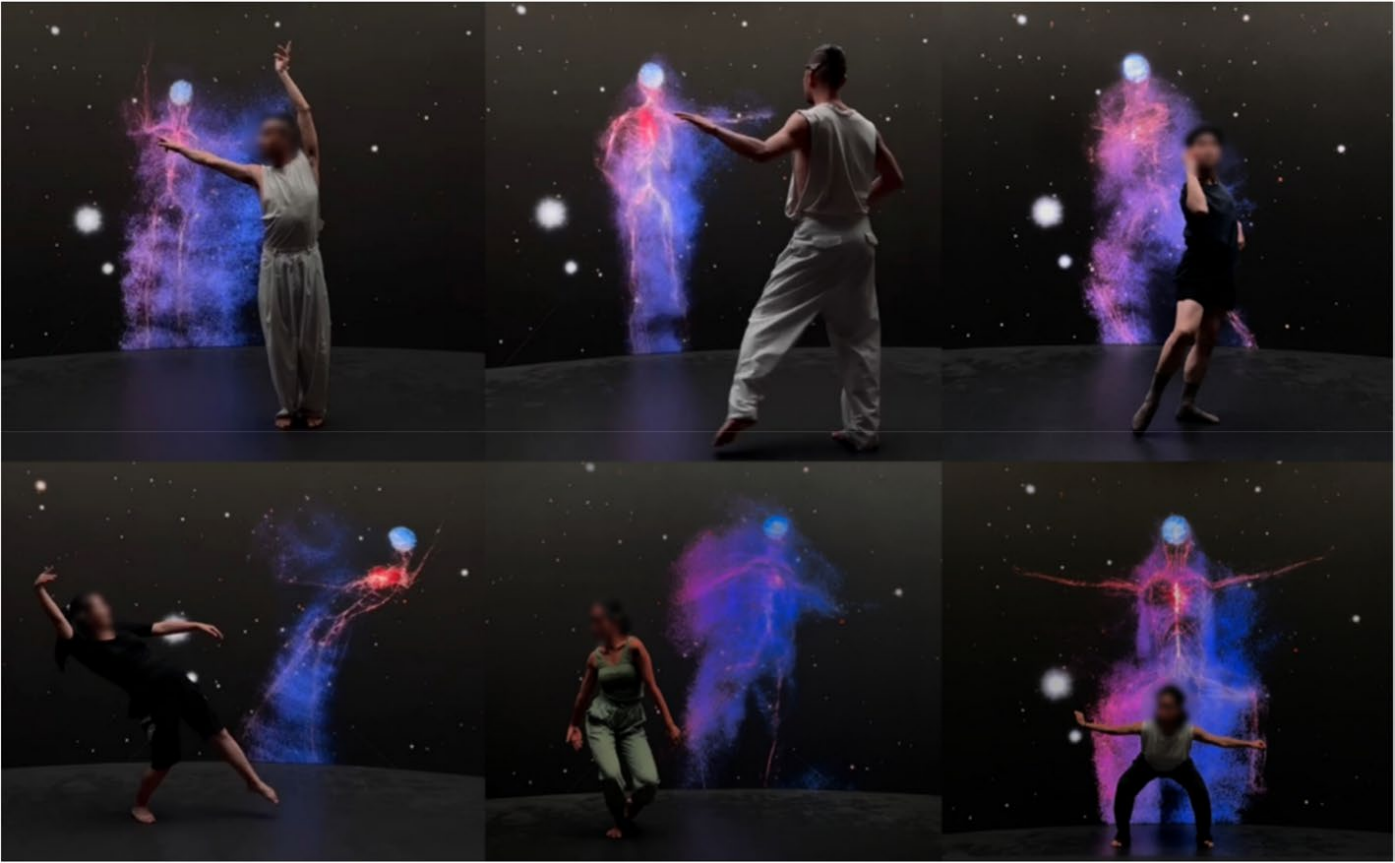
Dancing with your bio-body

Unlike other modes, dancing with your bio-body centers on external engagement (Fig. 8). In this setting, dancers interact with a digital counterpart–a bio-body that tracks and responds to their movements along the *z*-axis. This interaction transcends basic mirroring, incorporating responsive and layered feedback where the bio-body adjusts in real time to both physical gestures and internal states.

Such interplay dissolves the boundary between the dancer and the digital form, establishing a dynamic exchange instead of mere replication. It redefines traditional dance norms by encouraging performers to experience their bio-body as a co-creative agent or an embodied extension of themselves. Drawing from the notion of extended cognition [61], this experience demonstrates how thought and perception extend beyond the brain, through the body, and into its surrounding context.

The system operates through a technological framework that includes real-time motion tracking, biometric sensing, and digital visualization. These components work together to synchronize the dancer’s physical movements with immediate digital responses, effectively uniting the physical and virtual into a cohesive expressive medium.

### Performance-led research

#### Aim and study design

This study aims to evaluate the effectiveness of the “Body Cosmos 2.0” interface in enhancing dancers’ self-awareness, creativity, and expressive capabilities through embodied biofeedback. This study employed a performance-led research methodology [62] combined with structured surveys to collect quantitative data. This study was conducted in a controlled workshop with 24 dancers of varying levels of experience.

#### Ethics approval and consent to participate

This study was approved by our institutional review board (Protocol No. HKUST(GZ)-HSP-2024-0024) for human testing. All participants signed a consent form before participation and were informed about the data collection process.

EEG sensors were used to capture brainwaves, and heart rate monitors. They were assured that their data would remain anonymous and that they could withdraw from the study at any time without providing a reason.

#### Participants

The workshop included 24 dancers with varying levels of experience, ranging from beginners (less than 1 year) to professionals with more than 10 years of experience. The participants were balanced in terms of sex (10 male and 14 female participants) and provided a diverse sample for evaluating the interface’s effectiveness.

#### Materials and technologies

The “Body Cosmos 2.0” system utilized an EEG headband integrated with a heart rate sensor (brand: Flowtime) to capture real-time physiological data. Emotional indicators such as attention, relaxation, and stress levels were derived from these data and used to influence the system’s visual outputs. A VR headset (Oculus Quest 2) was employed to provide immersive experiences in the VR embodiment mode.

#### Study procedure

The workshop was conducted over two days, with each day structured around specific phases of interaction and evaluation.

On the first day, participants were introduced to “Body Cosmos 2.0”. This introduction covers the system’s functionality, the technologies involved (such as EEG sensors and heart rate monitors), and how their physiological data influences the system’s visual outputs. The participants then engaged in the VR Embodiment mode, where they explored their physiological states in a virtual, cosmic environment. This immersive experience allowed them to perceive their biodata in real time, fostering an initial connection with their physiological information. Following the VR experience, the participants completed the Post-Experience Survey, which captured their immediate reactions, changes in self-awareness of physiological data, and initial impacts on creativity and dance expression. Inspired by their VR experience, participants spent the remainder of the day collaboratively developing choreography that incorporated elements derived from their physiological data and the “Body Cosmos 2.0 interface.”

The second day focused on performance and evaluation. Participants performed the choreography they had developed on the first day, integrating the “Body Cosmos 2.0” interface into their dance practice. This performance provided an opportunity to explore the relationship between physical movement and digital representation. After the performance, participants completed a Post-Performance Survey, which evaluated the sustained effects of the system on their performance, deeper creative insights, and overall sense of connection to the environment.

#### Data analysis

Data were analyzed using structured survey responses. The pre- and post-workshop survey results were compared using descriptive statistics to identify trends and changes in participants’ self-reported measures. Responses on the Likert scale were summarized to calculate means and standard deviations, providing insights into the impact of the “Body Cosmos 2.0” system on dancers’ self-awareness, creativity, and physiological connection.

In addition to the surveys, qualitative data were gathered through direct observation, and discussions were facilitated with the participants. These qualitative insights were analyzed thematically, revealing prominent themes that emerged from the participants’ experiences. These qualitative findings complement the quantitative survey data, providing a deeper understanding of how “Body Cosmos 2.0” influences both the creative process and dance performance dynamics.

## Results and Discussion

This section presents the findings of a workshop conducted with 24 dancers, integrating quantitative survey data and qualitative insights gathered through direct observation and post-session discussions. The evaluation is divided into three phases.

- Pre-workshop survey: Established baseline measures of participants’ dance experiences, current connection to physiological data, and general creativity levels.
- Post-experience survey: Captured immediate reactions after engaging in the VR embodiment mode, focusing on changes in self-awareness of physiological data and initial creative impacts.
- Post-performance survey: Evaluate the sustained effects of the system on performance, deeper creative insights, and an overall sense of connection to the environment.

This structured approach ensures an understanding of how “Body Cosmos 2.0” affects dancers at different stages of interaction, from initial exposure to active performance.

This study was approved by the Institutional Review Board for Human Subject Testing. Participants signed a consent form before participating and were informed that all collected data would be analyzed anonymously. The participants were also informed that they could withdraw from the study at any time for any reason.

### Demographics and baseline measures

Among the 24 participants, their dance experience varied. Eight dancers reported having 5–10 years of experience, seven had more than 10 years, six had 2–5 years, and three had less than one year of experience.

Initial connection to the environment (question 1): Before the VR experience, the participants’ self-reported connection to the environment ranged from 1 to 5 on a Likert scale. The average score before the experiment was approximately 3.0, indicating a moderate baseline connection among the participants.

Understanding of life data (question 2): Initially, the participants had varying degrees of understanding of their physiological data. The average score was approximately 3.0, suggesting general awareness but limited in-depth understanding of metrics such as heart rate and EEG data.

### Impact of the “Body Cosmos 2.0” experience on dance and creativity

Post-experience, the “Body Cosmos 2.0” system demonstrated significant effects on various aspects of dancers’ practice and perception. The following subthemes encapsulate these impacts:

- Enhancement of life data understanding (question 6): The majority of dancers reported a significant deepening of their understanding of life data. Approximately 70% of the participants rated the enhancement as 4 or 5, indicating that “Body Cosmos 2.0” effectively increased their awareness and comprehension of their physiological states.
- Importance of life data in dance creation (question 3): Participants largely recognized the importance of life data in dance creation, with an average rating of 4.2 post-experience. This underscores the system’s role in highlighting the relevance of physiological metrics in enhancing artistic expressions.
- Helpfulness to dance creation (question 7): Approximately 65% of dancers rated life data visualization as highly helpful (ratings of 4 or 5) in their dance creation processes.
- Influence on dance performance (question 8): Approximately 60% perceived a substantial influence of the VR experience on dance performance.
- Enhancement of dance expression (question 9): The majority (75%) agreed that understanding and applying life data enhanced dance expression.
- Inspiration to creativity (question 10): The VR experience was rated highly for inspiring creativity, with 70% of the participants giving high ratings (4 or 5).
- Creativity levels (questions 4 and 10): After the experience, the participants reported an average increase in perceived creativity levels. The correlation between enhanced life data awareness and creativity suggests that “Body Cosmos 2.0” facilitates creative thinking by providing real-time physiological feedback, allowing dancers to explore new movement paradigms influenced by their internal states.
- Previous VR experience (question 5): Participants had varied levels of prior exposure to VR art, with an average of 2.5 previous VR experiences. This diversity in prior experience did not significantly skew the results, indicating that “Body Cosmos 2.0,” regardless of previous VR familiarity, is accessible and impactful.
- Connection to the environment post-experience (question 11): After engaging with “Body Cosmos 2.0,” the participants reported an average increase in their sense of connection to the environment, with most ratings shifting from moderate to high (4 or 5). This aligns with the system’s aim of bridging internal physiological states with external artistic expressions, fostering a more profound sense of interconnectedness.

### Qualitative insights from observations and discussions

In addition to the structured surveys, qualitative data were gathered through observations and discussions. The analysis of these insights reveals three key themes.

1. Tension between internal force and external distraction: Dancers reported a persistent conflict between their intrinsic drive to express internal states and their need to remain attentive to external stimuli. This tension influenced how participants navigated the performance space and engaged with the system’s visual outputs.
2. Feedback reinforcing positive *vs* negative feelings: Participants noted that real-time biofeedback sometimes amplified positive emotions, whereas in other cases, it seemed to reinforce negative feelings. This variability suggests that individual emotional baselines and contextual factors can affect feedback interpretation.
3. Passive response *vs* active manipulation of feedback: Observations revealed differences in how dancers engaged with the system. Some participants responded passively, allowing feedback on their movements without deliberate modification. In contrast, others actively manipulate their emotions and movements to produce the desired visual patterns effectively, using the system as a tool for expressive control.

### Discussion

The survey results demonstrated that “Body Cosmos 2.0” effectively enhanced dancers’ awareness and understanding of their physiological data, which in turn positively influenced their creativity and dance expression. The significant increase in the perceived connection to the environment post-experience suggests that the system serves as both a biofeedback tool and a medium for deeper existential and artistic exploration. These findings align with previous studies that have explored the intersection of biofeedback and artistic expression, highlighting the transformative potential of integrating physiological data into creative practices [2, 44].

Embodied biofeedback and creativity: The positive correlation between biodata awareness and creativity underscores the potential of embodied biofeedback systems to augment artistic processes. By visualizing real-time physiological states, “Body Cosmos 2.0” allows dancers to integrate their internal experiences into their movements, fostering a more holistic and expressive performance. This resonates with Varela’s theory of the embodied mind [46], which posits that cognition arises from dynamic interactions between the body and its environment. Moreover, similar to the findings of Gruzelier et al. [2], our study suggests that biofeedback can boost creativity and reduce anxiety, thereby fostering optimal performance in dancers.

Implications for HCI in dance: “Body Cosmos 2.0” exemplifies the transformative potential of the human-technology relationship in the performing arts. The system provides dancers with immediate feedback, enabling a dynamic and adaptive artistic process that evolves in real time alongside their physiological state. This aligns with the broader discourse in media theory on the role of technology as an extension of human capabilities [49]. Furthermore, the bio-body concept bridges the gap between the virtual and cyber-body, offering a fusion of biological data and digital technology that enhances both self-augmentation and insight [51, 53, 54].

Embodied cognition framework integration: The study’s findings are deeply rooted in the embodied cognition framework outlined in the conceptual sections. By leveraging real-time physiological data, “Body Cosmos 2.0” facilitates a continuous feedback loop where dancers’ movements influence and are influenced by their internal states. This bidirectional interaction exemplifies Merleau-Ponty’s notion of embodied perception [47], emphasizing the inseparability of perception and action. The enhanced self-awareness and creative expression observed in the participants reflect the efficacy of embodied biofeedback in fostering a more integrated cognitive and artistic experience.

### Limitations and future work

Although “Body Cosmos 2.0” demonstrates promise in enhancing dancers’ self-awareness, creativity, and expressive capabilities through embodied biofeedback, several limitations must be addressed to fully realize its potential.

Deriving K: Integrating K into biodata is an ambitious goal, but deriving the K algorithmically from movements and expressions remains challenging. Our current approach captures only a limited subset of the rich nuances inherent in artistic expressions. Future iterations should refine the K → E → P integration by incorporating additional qualitative data, advanced machine learning techniques, and multimodal inputs–such as environmental context and social interactions–to more accurately represent a dancer’s long-term artistic insights.

Comparative analysis of experience modes: The current study did not facilitate a comparative analysis of the three experience modes: “VR experience embodiment,” “dancing within your bio-body,” and “Dancing with your bio-body.” Future research should systematically compare these modes to determine which specific features most effectively foster self-awareness and creativity and guide targeted improvements to the system.

Scope of dance practices: This work focuses primarily on dance improvisation, a form that emphasizes spontaneous expression. Although this approach offers unprecedented insights, it does not encompass the full spectrum of dance practices. Future studies should explore the integration of choreographed pieces, structured frameworks, and partner work, thereby broadening the applicability of the system and deepening our understanding of its impact across diverse dance forms.

Depth of participant experience: The current evaluation relied mainly on self-reported survey data, which, while useful, may not capture the full depth of the participants’ experiences. Future studies should incorporate comprehensive qualitative methods such as interviews, focus groups, and detailed video analyses to gain deeper insights into participants’ motivations, challenges, and creative processes.

Objective data collection: The Reliance on self-reported measures introduces subjectivity and potential bias. Future iterations of this study should integrate objective physiological and performance metrics to corroborate self-reported outcomes and provide a more robust evaluation of the impact of the system.

## Conclusions

“Body Cosmos 2.0” represents an advancement in the integration of embodied biofeedback and dance, offering an interactive interface that bridges the gap between human physiology, the digital environment, and artistic expression. By introducing the concept of the “Bio-body,” this system enables dancers to engage with their physiological and emotional states in unprecedented ways, offering immersive experiences that enhance self-awareness, creativity, and performance.

Through its three distinct modes: “VR embodiment,” “dancing within your bio-body,” and “dancing with your bio-body,” “Body Cosmos 2.0” fosters a deeper connection between the mind, body, and digital representation. These modes provide dancers with a range of immersive interactions, from experiencing internal states from a first-person perspective to exploring new forms of collaboration with their digital counterparts. The unique integration of biofeedback data and artistic expressions transcends traditional neurofeedback, offering a deeper exploration of embodied cognition and expanding the creative potential of dance.

While the initial evaluation provided valuable insights into the system’s potential, further research is needed to address its limitations, refine the system, and expand its applicability. Comparative analyses of the different experience modes, incorporation of qualitative methods, and inclusion of objective physiological data will provide a more comprehensive understanding of how the “Body Cosmos 2.0” influences dancers’ creative processes and self-regulation. Additionally, future work will explore the customization of biofeedback P, longitudinal studies, and the application of the system across diverse dance genres and cultural contexts.

## Supplementary Information

Supplementary Material 1.

## Data Availability

Not applicable.

## Abbreviations

VR: Virtual reality
HCI: Human computer interaction
BCI: Brain-computer interface
SDK: Software development kit
EEG: Electroencephalography
CT: Computed tomography
DICOM: Digital imaging and communications in medicine
VDB: Voxel data block
OSC: Open sound control
A: Artistic visualization
C: Cognitive data
P: Parameters
K: Artistic knowledge
MRI: Magnetic resonance imaging
3D: Three-dimensional
