# Supplementary material for: Body Cosmos 2.0: embodied biofeedback interface for dancing
Source: Vis Comput Ind Biomed Art. 2025 Nov 20;8:26. doi: 10.1186/s42492-025-00207-9 (PMC12634995; doi:10.1186/s42492-025-00207-9)
Supplement: Supplementary file 1 — Supplementary Material 1. [file 42492_2025_207_MOESM1_ESM.docx]

**5 Appendix**

This section provides descriptions of key terms and technologies related to the research, including methodologies, tools, and systems used in areas such as neuroscience, VR, human-computer interaction, and digital media. These definitions clarify the technical vocabulary used in this study.

Electroencephalography (EEG): A technique for measuring electrical activity in the brain is typically used to monitor brainwave patterns such as attention, relaxation, and cognitive load.

Virtual reality (VR): A simulated environment created by computer systems that users can interact with, usually through immersive devices such as headsets and motion tracking.

Human computer interaction (HCI): The field of study and design focuses on how humans interact with computers and technology, particularly the usability and interface design aspects.

Brain-computer interfaces (BCIs) are systems that enable direct communication between the brain and external devices, facilitating the control of technology through neural signals.

Software development kits (SDKs): Collections of tools, libraries, and documentation provided to developers to create software applications for specific platforms or systems.

Digital imaging and communications in medicine (DICOM): A standard for the management, storage, and transmission of medical imaging data (e.g., X-rays, CT scans, and MRIs) to ensure interoperability across devices and platforms.

Computed tomography (CT) is a medical imaging technique that uses X-rays and computer processing to produce cross-sectional images of the body and is often used to diagnose conditions such as cancers and injuries.

Magnetic resonance imaging (MRI) is a noninvasive medical imaging method that uses magnetic fields and radio waves to generate detailed images of internal body structures such as organs and tissues.

Natural scenes dataset (NSD): A curated collection of real-world images is used in neuroscience and machine learning to analyze visual processing and neural responses to natural scenes.

Portable network graphics (PNG): A lossless image format commonly used for web graphics that supports high-quality detail, transparency, and a broad color range.

Voxel data block (VDB): A file format for storing volumetric data that is often used in 3D graphics and simulations to represent smoke, fluids, or other dynamic effects in computer-generated environments.

Houdini: 3D animation and visual effects software for creating complex simulations, procedural modeling, and digital content that is widely used in the film and gaming industries.

Vector expression language (VEX): This programming language is used by the company to create custom shaders and simulations, enabling high-performance calculations in a procedural context.

Unreal engine 5 (UE5): A high-performance game engine developed by Epic Games is used to create interactive media, simulations, and video games with advanced graphics and real-time performance.

Niagara system: A visual effects system in Unreal Engines 4 and 5 was designed to create complex particle simulations such as smoke, fire, and fluid dynamics, which are often used in gaming and cinematic applications.

Horizontal and vertical texture coordinates (UV): A system was used to map the 2D textures onto the 3D models, where U and V represent the horizontal and vertical axes of the texture, respectively.

Open sound control (OSC): A protocol for communication between multimedia devices and software that facilitates the exchange of sound, audio, and control data across networks.

User datagram protocol (UDP): This is a connectionless communication protocol that sends data packets quickly but with less reliability than TCP, which is commonly used in real-time applications such as video streaming and online gaming.
